# Clinical Efficacy of Vascular Sealant in Promoting Rapid Recovery After Total Thyroidectomy for Thyroid Cancer

**DOI:** 10.7759/cureus.91305

**Published:** 2025-08-30

**Authors:** Yu Cheng, Wangming Ji, Shuang Zhang, Zhiwei Liu, Lili Liu, Jianhua Gu, Yanming Wang

**Affiliations:** 1 Department of Thyroid, Breast and Hernia Surgery, Tianjin First Central Hospital, Tianjin, CHN; 2 Department of Hepatobiliary Surgery, Rockets Army General Hospital of People's Liberation Army (PLA), Beijing, CHN; 3 Department of Pediatrics, Tianjin Dongli Hospital, Tianjin, CHN; 4 Department of Plastic and Burn Surgery, Tianjin First Central Hospital, Tianjin, CHN; 5 Department of Medicinal Chemistry and Pharmaceuticals Science, College of Pharmacy, Key Laboratory of Bioactive Materials for the Ministry of Education, Nankai University, Tianjin, CHN

**Keywords:** drainage volume, enhanced recovery after surgery, neck drainage volume, total thyroidectomy for thyroid cancer, vascular sealant

## Abstract

Objective

To explicitly evaluate the clinical value of vascular sealant in enhancing early recovery following total thyroidectomy for thyroid cancer, with a specific focus on reducing perioperative complications, such as tissue fluid exudation, wound oozing, and lymph leakage.

Methods

A retrospective clinical study was conducted, involving 162 patients with thyroid cancer who were recruited at Tianjin First Central Hospital from January 2023 to December 2024. The cohort consisted of 62 men and 100 women, aged 20-78 years. Based on the method of wound management during surgery, patients were divided into the vascular sealant group (observation group, n=82) and the traditional wound management group (control group, n=80). Both groups underwent total thyroidectomy combined with bilateral central neck lymph node dissection. General clinical indicators such as operation time and intraoperative blood loss were recorded. The drainage volume on postoperative days 1 and day 2, total neck drainage during the perioperative period, and the duration of drain placement were compared between the two groups. Blood tests were conducted preoperative and on postoperative day 3 to assess white blood cell count, neutrophil count, C-reactive protein (CRP), and procalcitonin (PCT) levels. Perioperative complications were analyzed. Statistical analysis was performed using non-parametric tests for independent samples, chi-square tests, and other appropriate methods to evaluate the indices.

Results

On postoperative days 1 and day 2, the drainage volume, total neck drainage during the perioperative period, and the duration of drain placement in the observation group were significantly lower than those in the control group (all P<0.05). There were no significant differences in the preoperative white blood cell count, neutrophil count, CRP, and PCT levels between the two groups (all P>0.05). On postoperative day 3, the levels of these indicators were significantly lower in the observation group than in the control group (all *P<*0.05). Compared to their preoperative baseline, both groups showed significant increases in these indicators on postoperative day 3 (all P<0.01). The incidence of wound oozing (8, 9.8%) and lymph leakage (0, 0%) in the observation group was significantly lower than that in the control group ((17 (21.2%), 5 (5.0%)) (P<0.05).There was no significant difference in the risk of postoperative fever between the two groups (P>0.05).

Conclusion

Vascular sealant can effectively reduce tissue fluid exudation in the surgical area, decrease the risk of wound oozing and lymph leakage, and has significant advantages in promoting rapid postoperative recovery in patients undergoing total thyroidectomy for thyroid cancer.

## Introduction

Thyroid cancer (TC), as a common malignancy of the endocrine system, has surgical resection as the primary treatment option. However, the complex anatomical structure of the thyroid, along with its proximity to blood vessels and lymphatic vessels, results in a high risk of postoperative complications such as hemorrhage and lymphorrhea. Guidelines [1,2] indicate that the incidence of postoperative bleeding in TC is approximately 0.45% to 4.2%, and the incidence of lymph leakage after lateral neck lymph node dissection can be as high as 4.5% to 8.3%. Achieving effective intraoperative hemostasis and sealing of lymphatic vessels is essential for preventing complications and facilitating early recovery. Nevertheless, in traditional surgical techniques, methods such as electrocoagulation or suturing of blood vessels still have limitations in effectively reducing the risk of complications, thereby prolonging hospitalization and increasing medical costs. With the promotion of the enhanced recovery after surgery (ERAS) concept, how to accelerate the recovery process while ensuring treatment efficacy has become a focal point of research in thyroid surgery.

In recent years, the development of new hemostatic materials has provided a new direction for optimizing vascular handling. Among them, vascular sealant, as a bioactive fibrin adhesive, can quickly form a fibrin mesh at the surgical site to assist in hemostasis and seal lymphatic vessels. It has shown potential advantages in reducing perioperative infection risks, improving postoperative wound exudation, and enhancing perioperative recovery speed [3,4]. However, its application in thyroid surgery remains an area that requires further investigation. Therefore, this study aims to evaluate the efficacy of vascular sealant in reducing perioperative complications, including bleeding, lymph leakage, and exudation, while promoting a faster recovery post-total thyroidectomy for thyroid cancer. This will provide evidence-based support for the clinical application of vascular sealants in thyroid surgery.

## Materials and methods

General information

This study is a retrospective clinical study. A total of 162 patients with papillary thyroid cancer who required surgical treatment and were admitted to the Department of Thyroid Surgery at Tianjin First Central Hospital from January 2023 to December 2024 were selected as the study subjects. Patients were enrolled based on inclusion and exclusion criteria. All patients underwent total thyroidectomy and bilateral central neck lymph node dissection. Based on the method of wound management during surgery, the patients were divided into the observation group and the control group. The observation group included 82 patients, who had vascular sealant applied to the surgical site after surgery; the control group consisted of 80 patients, who underwent traditional wound management. There were no significant differences in baseline characteristics between the two groups (P>0.05). This study has been approved by the Ethics Committee of Tianjin First Central Hospital (Ethical Approval No.: 2022DZX02). All participants signed informed consent forms prior to the commencement of the study, fully understanding the research objectives, methods, potential risks, and their rights. The research process strictly adhered to the ethical principles outlined in the Declaration of Helsinki, and all collected clinical data were de-identified to ensure the privacy and data security of the participants.

Inclusion criteria

(1) Patients aged 18-80 years. (2) Tumor diameter in the thyroid isthmus >1.0 cm. (3) Non-thyroid isthmus tumors with a diameter <1.0 cm, with bilateral multifocal tumors. (4) Non-thyroid isthmus tumors with a diameter of 1.0-4.0 cm, accompanied by any of the following high-risk factors: thyroid capsule invasion, preoperative detection of central neck lymph node metastasis (≥five lymph nodes or ≥3 cm in diameter), a history of head and neck radiation exposure during adolescence, or any other factors requiring postoperative treatment. (5) Preoperative ultrasound elastography and fine needle aspiration pathological assessment show no lateral neck lymph node metastasis. (6) All patients were undergoing their first surgery and had not received prior treatment with radiofrequency or microwave ablation, ethanol injection, chemotherapy, or targeted therapy.

Exclusion criteria

(1) Patients with various advanced metabolic diseases; (2) patients with chronic renal failure who had already undergone dialysis treatment; (3) retrosternal thyroid goiter; (4) tumors invading or encircling the internal jugular vein or with distant metastasis; (5) patients with a history of malignancies other than thyroid cancer.

Surgical procedure

**Figure 1:**
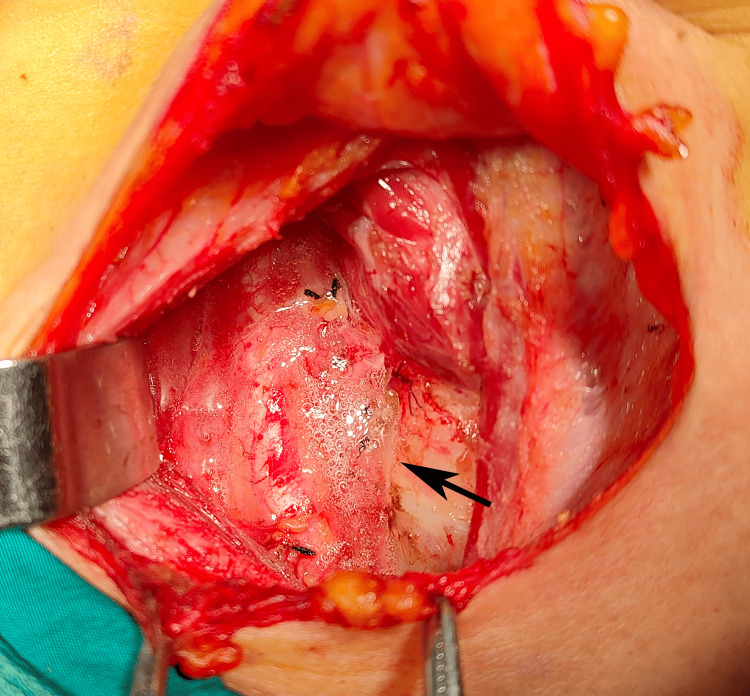
Vascular sealant sprayed on the surgical area The position of the black arrow indicates the site where the vascular sealant was sprayed, and fibrin adhesion was formed after solidification

All patients underwent total thyroidectomy combined with bilateral central neck lymph node dissection. During surgery, 3-0 silk sutures were used for ligation and hemostasis of major blood vessel ends, while bipolar electrocautery forceps were used for hemostasis of small blood vessels and wound oozing points. In the observation group, after completing the surgical procedure and confirming no active bleeding, the surgical site was cleaned of blood using dry gauze, and then absorbable vascular sealant (CeloSeal, Selexis Biotechnology Co., Ltd., Geneva, Switzerland; Medical Device Registration No.: 20193020081, 2mL specification) was applied. The sealant was prepared 15 minutes prior to use and evenly sprayed onto the surgical area (as shown in Figure 1). After the biological adhesive had solidified, one drainage tube was placed in the neck surgical site, connected to a disposable vacuum negative pressure drainage device Safevac (Medinorm, Spiesen-Elversberg, Germany, pre-set vacuum pressure=95 kPa). In the control group, after completing the surgical procedure, the surgical area was cleaned with dry gauze and the negative pressure drainage device of the same specification was placed at the surgical site. All surgical procedures were performed by a senior surgeon to minimize research bias.

Observational indicators

(1) Clinical indicators: The following general clinical indicators were recorded for both groups: operation time, intraoperative blood loss, and other related data. Operation time was recorded starting from the skin incision and continuing until the total thyroidectomy was completed, measured in minutes (min). Intraoperative blood loss was assessed by weighing the difference in the weight of the gauze before and after surgery, with each gram of weight increase corresponding to approximately 1 mL of blood absorbed.

(2) Postoperative recovery indicators: The following recovery indicators were analyzed for both groups: drainage volume on postoperative days 1 and day 2, total neck drainage during the perioperative period, and duration of drain placement. Neck drainage volume was calculated based on the total volume in the vacuum drainage bottle after surgery, and assessed in milliliters (mL). Drain placement time was calculated from the day of surgery to the day of drain removal, with drain removal being performed when the daily neck drainage volume was ≤15 mL. The results were expressed in days (d).

(3) Hematological inflammatory indicators: Blood samples (5 mL) were collected from the patients on the morning of both preoperative (baseline) day and postoperative day 3. The samples were analyzed using an automatic biochemical analyzer to assess the levels of white blood cell count, neutrophil count, C-reactive protein (CRP), and procalcitonin (PCT) in whole blood.

(4) Perioperative complication risks:Perioperative complications, including fever, wound oozing, and lymph leakage, were observed and recorded for both groups. Wound oozing was defined as the presence of dark red blood drainage from the surgical site within 24 hours after surgery, with blood clots present in the drainage bottle, and possibly accompanied by anterior neck swelling. Lymph leakage was defined as the appearance of milky, turbid fluid in the drainage tube or bottle after surgery.

Statistical analysis

Data analysis was performed using SPSS version 20.0 software (IBM Corp, Armonk, NY). For normally distributed continuous data, the results were expressed as mean±standard deviation (x̄±*s*), and inter-group comparisons were made using the t-test. Perioperative complication data, being categorical, were compared between groups using the chi-square (*χ²*) test. A P-value of <0.05 was considered statistically significant.

## Results

Analysis of general clinical data and postoperative recovery indicators for both groups

There were no statistically significant differences in the baseline data between the two groups (as shown in Table 1). Comparisons of operation time and intraoperative blood loss between the two groups showed no significant differences (t=1.499, 1.471, P=0.136, 0.143) (Table 2). On postoperative days 1 and day 2, the neck drainage volume and total neck drainage during the perioperative period were significantly lower in the observation group compared to the control group, with statistically significant differences (t=-10.336, -9.339, -32.582, all P<0.001). The duration of drain placement in the observation group was significantly shorter than in the control group, with a statistically significant difference (t=-8.350, P<0.001), as shown in Table 2.

**Table 1:** Analysis of Baseline Clinical Data of Both Groups TI-RADS: Thyroid Imaging Reporting and Data System; TNM: tumor, node, metastasis

Clinical indicators	Observation Group (n=82)	Control Group (n=80)	t(x^2^) value	P-value
Age (years,x±s)	49.72±12.50	48.76±13.68	0.465	0.643
Gender(cases)				
Male	34（41.5%）	28（35.0%）	（0.716）	0.397
Female	48（58.5%）	52（65.0%）		
Number of Tumors (cases)				
1	33（40.2%）	47（58.8%）	（5.629）	0.131
2	28（34.1%）	18（22.5%）		
3	12（14.6%）	9（11.2%）		
≥4	9（11.0%）	6（7.5%）		
Maximum Tumor Diameter (cm, x±s)	1.44±0.56	1.49±0.53	-0.524	0.601
TI-RADS Classification (cases)				
Ⅳa	26（31.7%）	33（41.2%）	（2.408）	0.492
Ⅳb	27（32.9%）	19（23.8%）		
Ⅳc	17（20.7%）	18（22.5%）		
Ⅴ	12（14.6%）	10（12.5%）		
Tumor Location (cases)				
Thyroid lobe (unilateral or bilateral)	70（85.4%）	65（81.2%）	（0.494）	0.482
Thyroid isthmus	12（14.6%）	15（18.8%）		
Ultrasound Evaluation of Lymph Node Metastasis (cases)				
0	25（30.5%）	29（36.2%）	（1.440）	0.696
1-3	31（37.8%）	24（30.0%）		
4-6	19（23.2%）	18（22.5%）		
≥7	7（8.5%）	9（11.2%）		
TNM Stage				
Ⅰ	55（67.1%）	55（68.8%）	（0.714）	0.700
Ⅱ	11（13.4%）	13（16.2%）		
Ⅲ	16（19.5%）	12（15.0%）		
History of Hypertension				
Yes	37（45.1%）	29（36.2%）	（1.320）	0.251
No	45（54.9%）	51（63.8%）		
History of Diabetes				
Yes	36（43.9%）	27（33.8%）	（1.756）	0.185
No	46（56.1%）	53（66.2%）		

**Table 2:** Comparison of General Clinical Data and Postoperative Recovery Indicators Between the Two Groups

Clinical indicators	Observation Group (n=82)	Control Group (n=80)	t-value	P-value
Operation Time (min)	87.80±7.33	86.03±7.78	1.499	0.136
Intraoperative Blood Loss (mL)	16.54±3.85	15.56±4.56	1.471	0.143
Postoperative Day 1 Drainage Volume (mL)	18.76±3.77	27.41±6.50	-10.336	<0.001
Postoperative Day 2 Drainage Volume (mL)	16.15±4.67	23.61±5.49	-9.339	<0.001
Total Neck Drainage During Perioperative Period (mL)	42.24±5.93	79.19±8.28	-32.582	<0.001
Duration of Drain Placement (days)	3.06±1.11	4.61±1.25	-8.350	<0.001

Evaluation of inflammatory indicators

At baseline, there were no statistically significant differences between the two groups in white blood cell count, neutrophil count, CRP, and PCT levels (t=0.592, 1.139, -0.857, -1.487, all P>0.05). On postoperative day 3, the levels of these indicators were significantly lower in the observation group compared to the control group, with statistically significant differences (t=-5.001, -4.914, -2.622, -3.371, all P<0.05), as shown in Table 3. In both the observation and control groups, the levels of these indicators on postoperative day 3 were significantly higher than their respective baseline levels, and the inter-group differences were statistically significant (t_observation group_=-4.774, -15.009, -27.457, -16.348;t_control group_=-8.889, -19.536, -29.912, -17.232, all P<0.01).

**Table 3:** Evaluation of Perioperative Inflammatory Indicator Changes in Both Groups CRP: C-reactive protein; PCT: procalcitonin; a indicates comparison of the observation group’s postoperative day 3 indicators with preoperative values, all P<0.01; b indicates comparison of the control group’s postoperative day 3 indicators with preoperative values, all P<0.01.

Inflammatory Indicators	Observation Group (n=82)	Control Group (n=80)	t-value	P-value
Preoperative (Baseline)				
White Blood Cell Count (*10^^9^/L)	6.81±1.20	6.69±1.40	0.592	0.555
Neutrophil Count (*10^^9^/L)	4.79±1.05	4.62±0.89	1.139	0.256
CRP（mg/L）	1.44±0.49	1.51±0.62	-0.857	0.393
PCT（ng/mL）	0.027±0.012	0.029±0.011	-1.487	0.139
Postoperative Day 3				
White Blood Cell Count (*10^^9^/L)	7.76±1.45^ a^	8.86±1.34^ b^	-5.001	<0.001
Neutrophil Count (*10^^9^/L)	7.02±1.18^ a^	7.97±1.28^ b^	-4.914	<0.001
CRP（mg/L）	11.67±3.38^ a^	13.07±3.39^ b^	-2.622	0.010
PCT（ng/mL）	0.214±0.103^ a^	0.276±0.130^ b^	-3.371	0.001

Perioperative complications

There was no statistically significant difference in the risk of postoperative fever between the two groups (*χ²*=0.375, P=0.540). The incidence of wound oozing (8, 9.8%) and lymph leakage (0, 0%) in the observation group was significantly lower than that in the control group (17 (21.2%), 5 (5.0%)), with statistically significant differences between the groups (*χ²*=4.099, 5.204, P=0.043, 0.040), as shown in Table 4.

**Table 4:** Analysis of Perioperative Complications in Both Groups

Complications	Observation Group (n=82)	Control Group (n=80)	c^2^-value	P-value
Fever	6（7.3%）	4（5.0%）	0.375	0.540
Wound oozing at the surgical site	8（9.8%）	17（21.2%）	4.099	0.043
Lymph leakage	0（0%）	5（5.0%）	4.204	0.040

## Discussion

With the significant increase in the detection rate of thyroid tumors, the number of surgeries has been steadily rising each year. Postoperative bleeding and airway compression caused by bleeding are the most common early complications, and in severe cases, they can lead to asphyxiation and death, prolonging hospital stays and potentially causing medical disputes. Additionally, tissue fluid exudation at the surgical site and lymph leakage are key factors that limit rapid patient recovery [5].

Previous clinical studies have indicated that in patients with Hashimoto's thyroiditis, due to abundant blood supply and changes in thyroid texture, both postoperative blood loss and exudation are significantly increased. Valsalva maneuvers during the anesthesia recovery process, as well as severe coughing and vomiting during the postoperative recovery period, can cause a sharp increase in pressure in the neck and chest blood vessels. This pressure may result in the rupture of small blood vessels and lymphatic vessel ends that have been ligated, sealed, or are too small to be ligated, ultimately leading to bleeding or lymph leakage [6,7]. Although various energy platforms (such as bipolar electrocautery and ultrasonic scalpel) have been introduced to aid in the delicate dissection during surgery and have shown some success in hemostasis and reducing wound exudation, there are still limitations when dealing with complex vascular and lymphatic systems. Therefore, exploring methods to further reduce perioperative bleeding and exudation at the neck surgical site is crucial for improving patient quality of life and recovery speed.

The results of this study demonstrated that the application of vascular sealant effectively reduces drainage volume and postoperative complications, supporting its proposed mechanism of action through the formation of a fibrin matrix that seals blood vessels and lymphatic channels. This advantage is particularly evident in thyroid surgeries, where conventional hemostatic techniques may be insufficient in managing the complex vascular and lymphatic anatomy [8]. Additionally, the fibrinogen and thrombin active centers in the fibrin network can promote platelet aggregation and the activation of clotting factors, thereby accelerating the blood coagulation process [9]. Previous studies have shown that fibrin networks not only create a "barrier" at the wound site to prevent blood and tissue fluid leakage but also provide support for fibroblasts and new blood vessels through their mesh structure, promoting cell migration and proliferation, which accelerates wound repair and healing. This is particularly beneficial in surgeries involving areas with rich vascular and lymphatic systems [10,11]. The results of this study indicate that, while there were no significant differences in surgery time and intraoperative blood loss between the two groups, the neck drainage volume on postoperative days 1 and 2, and total neck drainage during the perioperative period in the observation group were significantly lower than in the control group. Furthermore, the incidence of wound oozing at the thyroid surgical site was also lower in the observation group, suggesting that vascular sealant is superior to traditional hemostatic methods in improving wound exudation and hemostasis.

Lymph leakage is a common complication after total thyroidectomy for thyroid cancer, especially during central neck lymph node dissection, as damage to lymphatic vessels can lead to the leakage of chylous fluid. Increased drainage volume can result in volume depletion, electrolyte imbalance, and hypoproteinemia [12]. The results of this study, based on the analysis of perioperative complications, showed that the incidence of lymph leakage was lower in the observation group compared to the control group. Curcio et al. [13] suggested that spraying vascular sealant rapidly forms a waterproof protective layer at the wound site, sealing the lymphatic vessel openings and reducing the leakage and loss of lymph fluid. This sealing effect not only prevents fluid leakage but also reduces dead space in the surgical area, limiting bacterial proliferation and further lowering the risk of infection. At the same time, the observation group showed a significantly shorter duration of drain placement compared to the control group. These findings collectively suggest that vascular sealant can effectively reduce postoperative blood and tissue fluid exudation at the thyroid and neck surgical sites, lower the risk of lymph leakage, shorten the duration of drain placement, and promote early recovery in patients.

In this study, although the white blood cell count, neutrophil count, CRP, and PCT levels were elevated compared to baseline levels in both groups, the levels of these indicators were lower in the observation group than in the control group on postoperative day 3. White blood cell and neutrophil counts are natural indicators of postoperative inflammation, and the elevation of CRP and PCT also suggests the presence of inflammation, which is considered a natural physiological response to surgical trauma and the recovery process, related to enhanced immune activity. Some studies have suggested that the main components of vascular sealant, fibrin and bioactive molecules, have good biocompatibility and play an important role in reducing postoperative inflammation [14,15]. Jia Teng et al. [16] pointed out that the physical barrier formed by the fibrin structure can prevent the wound from being exposed to external environmental irritants, thereby reducing the recruitment and activation of inflammatory cells. The results of this study, combined with previous research, suggest that vascular sealant has relatively low immunogenicity and may reduce the inflammatory response at the surgical site through its fibrin adhesion and sealing effect, further confirming its safety in clinical application.

Although this study preliminarily confirmed the positive effect of vascular sealing glue in promoting postoperative recovery following radical surgery for thyroid cancer, several limitations should be acknowledged. First, this was a single-center retrospective clinical study, which may have been subject to selection bias in patient enrollment. Therefore, the generalizability of the results remains to be validated through large-scale, multi-center prospective studies. Second, the study primarily focused on short-term postoperative recovery indicators, such as drainage volume, duration of drainage tube retention, and early inflammatory markers. However, it lacked a systematic assessment of long-term outcomes, including recurrence rate, quality of life scores, and postoperative functional recovery, which limits the comprehensive evaluation of the long-term efficacy of the intervention.

## Conclusions

The application of vascular sealant in total thyroidectomy for thyroid cancer not only improves the hemostasis and sealing effects of thyroid and neck surgical sites, effectively reducing tissue fluid exudation and the incidence of lymph leakage, but also shortens the duration of drain placement. These findings strongly support the clinical utility and advantages of vascular sealant as an adjunctive measure to facilitate enhanced recovery in thyroid cancer surgery. The observed outcomes are highly consistent with the principles of enhanced recovery after surgery (ERAS) and suggest that vascular sealant may offer an effective strategy to promote rapid postoperative rehabilitation in thyroid procedures.
